# Efficacy and Safety of Anti-PD-1/ PD-L1 Monotherapy for Metastatic Breast Cancer: Clinical Evidence

**DOI:** 10.3389/fphar.2021.653521

**Published:** 2021-06-29

**Authors:** Yihang Qi, Lin Zhang, Zhongzhao Wang, Xiangyi Kong, Jie Zhai, Yi Fang, Jing Wang

**Affiliations:** ^1^ Department of Breast Surgical Oncology, National Cancer Center/National Clinical Research Center for Cancer/Cancer Hospital, Chinese Academy of Medical Sciences and Peking Union Medical College, Beijing, China; ^2^ School of Population Medicine and Public Health, Chinese Academy of Medical Sciences and Peking Union Medical College, Beijing, China; ^3^ Melbourne School of Population and Global Health, The University of Melbourne, Melbourne, VIC, Australia; ^4^ Centre of Cancer Research, Victorian Comprehensive Cancer Centre, Melbourne, VIC, Australia

**Keywords:** PD-1, PD-L1, immunotherapy, breast cancer, safety, efficacy

## Abstract

**Background:** Success has been reported in PD-1/PD-L1 blockade *via* pembrolizumab, atezolizumab, or avelumab monotherapy in manifold malignancies including metastatic breast cancer. Due to lack of large-scale study, here we present interim analyses to evaluate the safety and efficacy of these promising strategies in patients with advanced breast cancer.

**Methods:** Six studies including 586 advanced breast cancer patients treated with anti-PD-1/PD-L1 monotherapy agents before July 1, 2020, were included. The anti-PD-1/PD-L1 agents include pembrolizumab, atezolizumab, land avelumab. Statistics was analyzed by R software and IBM SPSS Statistics 22.

**Results:** Global analysis showed that for this monotherapy, the complete response was 1.26%, partial response was 7.65%, objective response rate (ORR) was 9.85%, and disease control rate (DCR) was 18.33%. 1-year overall survival rate and 6-month progression-free survival rate were 43.34 and 17.24%. Overall incidence of adverse events (AEs) was 64.18% in any grade and 12.94% in severe grade, while the incidence of immune-related AEs (irAEs) was approximately 14.75%: the most common treatment-related AEs of any grade that occurred in at least 5% of patients were arthralgia and asthenia; the most common severe treatment-related AEs occurred in at least 1% of patients were anemia and autoimmune hepatitis; the most common irAEs were hypothyroidism. Besides, the incidence of discontinue and death due to treatment-related AEs was about 3.06 and 0.31%, respectively. Additionally, by comparing efficacy indicators between PD-L1–positive and PD-L1–negative groups, an implicated correspondence between efficacy and the expression of PD-L1 biomarker was found: the PR was 9.93 vs 2.69%; the ORR was 10.62 vs. 3.07%; the DCR was 17.95 vs. 4.71%.

**Conclusion:** Anti–PD-1/PD-L1 monotherapy showed a manageable safety profile and had a promising and durable anti-tumor efficacy in metastatic breast cancer patients. Higher PD-L1 expression may be closely correlated to a better clinical efficacy.

## Introduction

The latest data showed that up to 30% of women with non-metastatic, early-stage breast cancer at first diagnosis will finally develop distant metastatic disease and up to 6% of women diagnosed with breast cancer in the United States have metastatic disease at the time of their first visit (Gennari et al., 2005; Chia et al., 2007; Dafni et al., 2010; Waks and Winer, 2019). Although there is promising development in the treatment of metastatic breast cancer (MBC) and an encouraging median overall survival time approaching to 24 months (vary from months to years), known as an incurable and fatal disease subtype, advanced breast cancer still poses a vitally significant threat to the wellness of women all over the world (Greenberg et al., 1996; Kiely et al., 2011), making it a pressing priority to discover novel and effective approaches to improve overall survival and prognosis of patients who suffered from this dangerous disease.

With the goal of being an individualized and tailored approach, the therapeutic strategy of MBC depends on both clinical factors and tumor biology, including systemic medical treatment consisting of chemotherapy, endocrine therapy, and/or biologic therapies, and supportive care measures (Beslija et al., 2003; Pagani et al., 2010). For metastatic triple-negative breast cancer (mTNBC) patients (whose median OS is 8–13 months, accounting for 15–20% of breast cancer patients) (Wahba and El-Hadaad, 2015; Den Brok et al., 2017), there is no approved targeted treatment available, other than the administration of cytotoxic chemotherapy with a poor clinical outcome (Dent et al., 2007; Anders et al., 2016; Wang et al., 2019). Thus, novel and effective therapies are urgently needed for MBC especially for mTNBC patients who suffering from progressed disease even following standard therapies.

Immunotherapy comprises a promising and tolerable new area in breast cancer therapy, and immune checkpoint blocking is one of the most extensively used active immunotherapy. Excessive activation of T cells can be prevented by immune checkpoint, which is function as a protective molecule in immune system of human, subsequently maintaining self-tolerance and reduce excessive auto-immune reactions (Loibl et al., 2021). Immune checkpoint molecules are reported to be overexpressed in malignancy cells and facilitate tumor cells to escape immune surveillance and killing by human immune system. Programmed death-1 (PD-1) and inhibitors of its ligand PD-L1 are the most widely used checkpoint inhibitors. PD-1 is an inhibitory *trans*-membrane receptor expressed on the surface of immune effect cells including B cells, T cells, DCs, NK cells, and many TILs, and two ligands of PD-1 called PD-L1 and PD-L2 have been identified (Wang et al., 2019). Induced by the regulation of tumor-derived IL-18, PD-1 is expressed on immunosuppressive CD16 (dim) CD56 (dim) NK cells (Tang et al., 2018). By combining with the B7 globulin family ligand PD-L1, PD-1 is activated and subsequently limiting T-cell activity, prohibiting over-active autoimmunity and immunity (Ademuyiwa et al., 2012; Liu et al., 2013). Such interactions function differently in normal tissues and tumor cells: in normal tissues, by restricting inflammatory activities during infections, PD-1/PD-L1 interactions exert protection effect against excessive tissue damage (Wang et al., 2019); in tumor cells, such interaction resulted in immune exhaustion and downregulation of immune response (Loibl et al., 2021). PD-1/PD-L1 interactions were also reported in breast cancer, and the expression of PD-1/PD-L1 was confirmed to be closely related to the effect of checkpoint inhibitors (Mittendorf et al., 2015; Salatino et al., 2018; Zhu et al., 2018). By comparing the expression of PD-L1 in TNBC and other breast cancers from the transcriptional as well as protein aspect, Mittendorf et al. reported that 20% of TNBC patients expressed PD-L1, which was higher than other breast cancer. Additionally, they illustrate the phenomenon that a higher expression of PD-1 or PD-L1 had correlation with increased Foxp3+ Treg infiltration, which supported that the expression of PD-L1 was positively associated with malignant degree in TNBC (Mittendorf et al., 2015; Raghavendra et al., 2018).

In 2011, by targeting either the PD-1 receptor or its ligand PD-L1, these immune checkpoint inhibitors were first approved by the Food and Drug Administration (FDA) for applying to advanced metastatic melanoma. Inhibitors targeting PD-1 or PD-L1 were confirmed to exert objective, durable, and promising responses in patients with melanomas, renal cell carcinomas, and NSCLCs (Carreno et al., 1950; Sasidharan Nair and Elkord, 2018). Moreover, with the emerging of a large number of research works about PD-L1/PD-1 inhibition and breast cancer, preliminary data from several qualified clinical trials presented promising outcomes for patients with advanced stage/metastatic breast cancer. In this meta-analysis study, we conducted the present available data of several qualified studies to explore the efficacy and safety of anti–PD-1/PD-L1 monotherapy for MBC.

## Methods

### Literature Searches

We searched PUBMED, Embase, Cochrane, and www.clinicaltrials.gov databases to determine eligible studies from database inception to July 1, 2020. With no restriction on language, the strategy we used was as follows (anti-PD-1 OR anti-PD-L1 OR nivolumab OR pembrolizumab OR atezolizumab OR avelumab OR BMS-936559 OR durvalumab) AND (metastatic OR advanced) AND breast cancer. All words available for Medical Subject Headings (MeSH) were searched by MeSH. Conference abstracts before July 1, 2020 were also searched. Two investigators (YQ and LZ) independently screened the titles and abstracts of identified articles. Major conflicts were resolved by another two researchers (PB and XK). The full texts of identified studies were further reviewed by two independent reviewers (JW and YF). The search was again extended by review of references of articles included in the final selection.

### Selection Criteria

Eligible studies included in our meta-analysis were required to meet the following criteria: 1) prospective clinical trials; 2) patients were diagnosed with advanced/metastatic breast cancer that were treated with anti–PD-1/PD-L1 agents monotherapy; 3) oncologic therapy prior to anti–PD1/PD-L1 treatment was acceptable; 4) studies reporting data regarding the efficacy or safety of this therapy; 5) research report–specific data related response rate (WHO Criteria) and adverse events (AEs); and 6) studies limited to humans.

The exclusion criteria were 1) non-oncologic patients treated with anti–PD-1/PDL1 agents; 2) oncologic patients treated with anti–PD-1/PDL1 agents combined with other treatments simultaneously; 3) letters, case reports, reviews, conference proceedings, commentaries, quality-of-life studies, cost-effectiveness analyses, and publications in which the cancer data or immunotherapy data that could not be ascertained; and 4) duplicate publications or unpublished studies.

### Data Extraction

The titles and abstracts of all studies retrieved were independently reviewed by two authors. Then, the full texts of all potentially eligible studies were assessed. A standardized, pre-piloted form was used to extract relevant information from the included studies. The efficacy outcomes were complete response (CR); partial response (PR); objective response rate (ORR); and disease control rate (DCR) according to Version 1.1 of the Response Evaluation Criteria in Advanced Solid Tumors version (RECIST version). PD-L1–positive expression status was defined as PD-L1 expressed in ≥1% tumor cells or/and tumor-associated immune cells. The primary safety outcomes were detail adverse events and incidence of adverse events (AEs), immune-related AEs (irAEs), and their grade (1–5; recorded according to Version 4 of the Common Terminology Criteria for Adverse Events of the National Cancer Institute, CTC for AE version). Grades ≥3 were evaluated as severe or high grade. The secondary outcome was incidence of death due to treatment related AEs. Any discrepancies or problems arisen were solved by our discussion. Missing data were acquired and ascertained from the principal investigator by e-mails.

### Quality Assessment

The risk of bias for the studies was assessed by two independent investigators according to the Cochrane Risk of Bias Tool and used a weighted Cohen’s kappa coefficient (κ) to measure agreement (Higgins et al., 2011). We assessed the following components: sequence generation, blinding, allocation concealment, completeness of outcome data, incomplete outcome data, and other types of bias. Problems and disagreements were resolved by our discussion until we reached an agreement.

### Statistical Analysis

The CR, PR, ORR, DCR, and incidence of AEs, irAEs, and severe AEs were pooled and estimated for the included studies in this meta-analysis. Heterogeneity between studies was assessed by Q test and I^2^ statistics. If the I^2^ value was less than 50%, the meta-analysis was performed using the fixed effects model. Otherwise, the random-effects model was selected. Potential publication bias was examined by Egger’s test. Incidence was calculated using R software [R version 3.5.2] with package Meta function. RR was calculated using IBM SPSS Statistics 22.

## Results

### Literature Search

**FIGURE 1:**
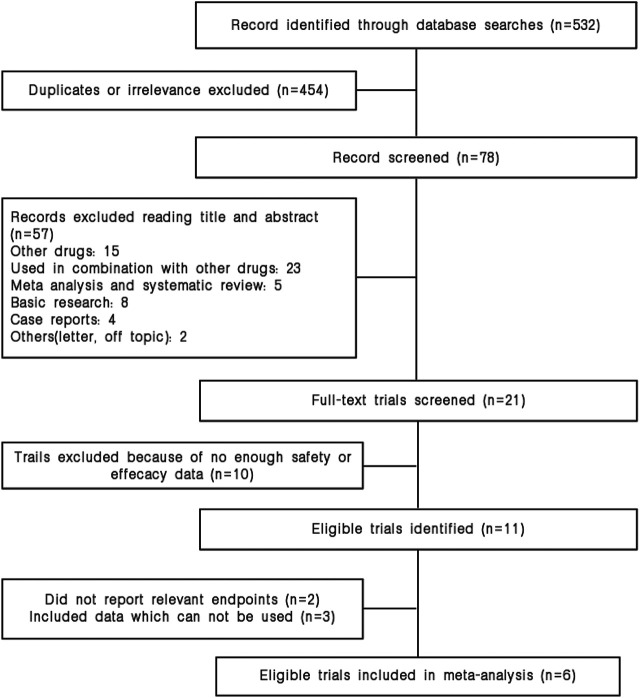
Flow diagram for identification and selection of studies included in the meta-analysis.

Our search strategy identified and reviewed 532 potential articles. 454 studies were excluded due to duplicates. The remaining 78 articles were screened for titles and abstracts, and 57 articles were removed based on our inclusion or exclusion criteria. Furthermore, fifteen studies were dropped because they did not contain our data of interest. Finally, six studies were included in our meta-analysis. The study selection is shown in Figure 1.

### Study Characteristics

The detailed information of involved clinical trials is presented in Table 1. Overall, six studies comprising 586 advanced breast cancer patients received anti-PD-1/PD-L1 monotherapy agents were included in this meta-analysis (Nanda et al., 2016; Dirix et al., 2018; Rugo et al., 2018; Adams et al., 2019; Emens et al., 2019) (Table 1). Among these studies, five studies were phase I clinical trials, and the other one was phase II clinical trial. All of them were single group assignment. There were four studies reporting pembrolizumab treatment (306 patients), one trial concerning atezolizumab treatment (115 patients), and the other one regarding avelumab treatment (168 patients). Among these 586 patients, 454 patients were TNBC, and 417 patients were PD-L1+ (PD-L1–positive status was defined as PD-L1 expressed in ≥1% tumor cells or/and tumor associated immune cells).

**TABLE 1:** Characteristics of clinical trials included for meta-analysis.

Study	NCT number	Title	Status	Conditions	Interventions	Characteristics					Population			Follow-up time frame	CTC for AE version	RECIST version
						Study type	Phase	Study design	Outcome measures	PD-L1 expression assessment	Enrollment	Age	Sex			
Emens, 2018	NCT01375842	A study of atezolizumab (an engineered anti-programmed death-ligand 1 [PDL-1] antibody) to evaluate safety, tolerability, and pharmacokinetics in participants with locally advanced or metastatic solid tumors	Completed	• Breast (advanced TNBC) • Tumors • Hematologic malignancies	• Biological: atezolizumab (15 or 20 mg/kg, or at a 1200-mg flat dose, every 3 weeks)	Interventional	Phase 1	• Allocation: randomized • Intervention model: parallel assignment • Masking: none (open label) • Primary purpose: treatment	• Number of participants with dose-limiting toxicities (DLTs) • Maximum tolerated dose (MTD) of atezolizumab • Recommended phase 2 dose (RP2D) of atezolizumab • Percentage of participants with adverse events •Percentage of participants with Anti-therapeutic antibodies (ATAs) • Area under the concentration–time curve (AUC) of atezolizumab etc.	Using the Ventana SP142 immunohistochemistry assay (Ventana Medical Systems), baseline of PD-L1 expression on ICs was evaluated with 4 scoring bins IC3 (≥10%), IC2 (5–10%), IC1 (≥1%,) and IC0 (<1%). The PD-L1 expression on tumor cells (TCs) was assessed as less than 1% (TC0) or at least 1% (TC1/2/3)	661 (115 breast cancer patients)	18 years and older (adult, older adult)	All	Up to 84 months	4	1.1
Adams, 2018	NCT02447003	Study of pembrolizumab (MK-3475) monotherapy for metastatic triple-negative breast cancer (MK-3475–086/KEYNOTE-086)	Active, not recruiting	Breast cancer (advanced TNBC)	• Biological: pembrolizumab (200 mg every 3 weeks)	Interventional	Phase 2	• Intervention model: single group assignment• Masking: none (open label) • Primary purpose: treatment	• Overall response rate (ORR) • Number of participants experiencing at least one adverse event (AE)• Number of participants discontinuing study drug due to AEs • Duration of response (DOR) • Disease control rate (DCR) • Progression-free survival (PFS) • Overall survival (OS) etc.	PD-L1 expression was assessed during screening using the PD-L1 IHC 22C3 pharmDx kit (Agilent, Carpinteria, CA, United States of America). The measure of expression was the combined positive score (CPS), defined as the ratio of PD-l1–positive cells (tumor cells, lymphocytes, and macrophages) out of the total number of tumor cells *100. PD-L1 positivity was defined as CPS ≥1 (previously reported as and equivalent to CPS ≥1%)	285 (all are breast cancer patients)	18 years and older (adult, older adult)	All	Up to 24–27 months	4	1.1
Rugo, 2018	NCT02054806	Study of pembrolizumab (MK-3475) in participants with advanced solid tumors (MK-3475–028/KEYNOTE-2	Active, not recruiting	• Solid tumor (including advanced breast cancer, ER + HER2-)	• Biological: pembrolizumab (10 mg/kg every 2 weeks)	Interventional	Phase 1	• Intervention model: single group assignment• Masking: none (open label) • Primary purpose: treatment	• Best overall response using response. Evaluation criteria in solid tumors (RECIST version 1.1) • Progression-free survival (PFS) • Overall survival (OS) • Duration of response (DOR) in participants who achieve partial response (PR) or better, etc.	Tumor PD-L1 expression was assessed by immunohistochemistry at a central laboratory using a prototype assay (QualTek molecular laboratories, Goleta, CA, United States of America) (28) and the 22C3 antibody (Merck and co, Kenilworth, NJ, United States of America). PD-L1 expression was determined by combined positive score (CPS), defined as the number of PD-l1–positive cells (tumor cells, lymphocytes, and macrophages) divided by the total number of tumor cells, multiplied by 100. The specimen is considered to have positive PD-L1 expression when CPS ≥1	477 (25 breast cancer patients)	18 Years and older (adult, older adult)	All	Up to 24 months	4	1.1
Dirix, 2017	NCT01772004	Avelumab in metastatic or locally advanced solid tumors (JAVELIN solid tumor)	Active, not recruiting	• Breast cancer (advanced BC) • Solid tumor	• Biological: avelumab (10 mg/kg every 2 weeks)	Interventional	Phase 1	• Intervention model: single group assignment • Masking: none (open label) • Primary purpose: treatment	• Dose-limiting toxicity and treatment-emergent adverse events • Confirmed best overall response (BOR) • Immune-related best overall response (irBOR) and best overall response (BOR) • Overall survival time (OS) and progression-free survival (PFS) time • Level of PD-L1 tumor expression, etc.	Levels of PD-L1 protein were assessed by immunohistochemistry using a proprietary assay (PD-L1 IHC 73–10 pharmDx; Dako, Carpinteria, CA, United States of America) with an anti–PD-l1 rabbit monoclonal antibody. Expression was based on the percentages of tumor cells expressing PD-L1: 1 and 5% thresholds with any staining intensity and a 25% threshold with moderate to high staining. Additionally, dense aggregates of tumor-associated immune cells (identified as nonmalignant cells based on morphology) adjacent to tumor cells were assayed using a defined threshold of 10% of immune cells expressing PD-L1 at any staining intensity	1758 (168 breast cancer patients)	18 years and older (adult, older adult)	All	Up to 52 months	4	1.1
Nanda, 2016	NCT01848834	Study of pembrolizumab (MK-3475) in participants with advanced solid tumors (MK-3475–012/KEYNOTE-01	Active, not recruiting	• Breast cancer (advanced TNBC) • Solid tumor	• Biological: pembrolizumab (10 mg/kg every 2 weeks)	Interventional	Phase 1	• Allocation: nonrandomized • Intervention model: parallel assignment • Masking: none (open label) • Primary purpose: treatment	• Number of participants experiencing adverse events (AEs) • Number of participants discontinuing from study treatment due to an AE • Overall response evaluation criteria in solid tumors version 1.1 (RECIST1.1) response rate based on blinded independent central radiology (BICR) Review (cohorts A, B, and B2, C, and D) • Overall RECIST 1.1 response rate based on investigator assessment for cohorts A, B, C, D	PD-L1 was assessed in formalin-fixed, paraffin-embedded archival tumor samples at a central laboratory using a prototype immunohistochemistry assay and the 22C3 antihuman PD-1 antibody (Merck and Co., Kenilworth, NJ).24 positivity was defined as PD-L1 expression in the stroma or in ≥1% of tumor cells	297 (111 breast cancer patients)	18 years and older (adult, older adult)	All	Up to 31–34 months	4	1.1

CTC for AE version, Common Terminology Criteria for Adverse Events version.

RECIST version, Response Evaluation Criteria in Advanced Solid Tumors version.

TNBC, triple-negative breast cancer; ER+, estrogen receptor‒positive; HER2−, human epidermal growth factor receptor 2 negative.

### Efficacy

#### Global Response Rate and Survival Rate

**FIGURE 2:**
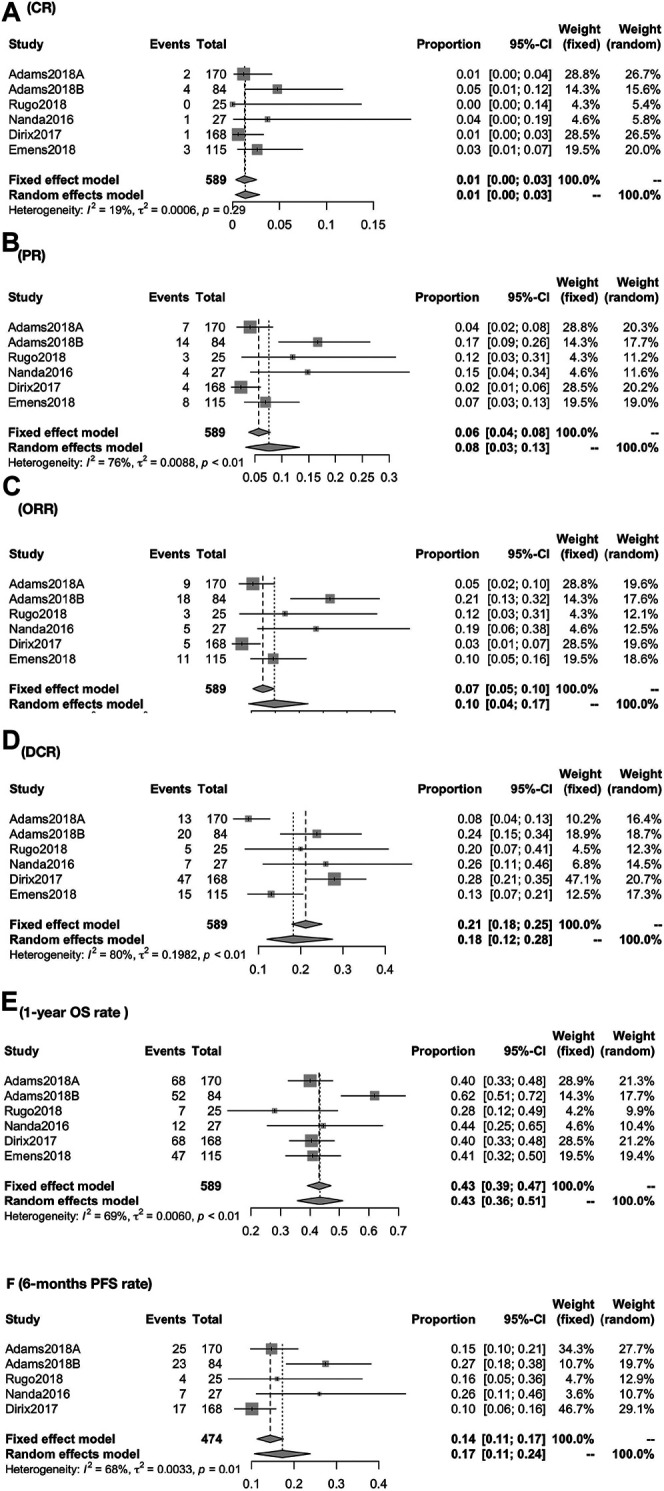
Global response rate for patients of CR **(A)**, PR **(B)**, ORR **(C)**, DCR **(D)**, 1-year OS rate **(E),** and 6-months PFS rate **(F)**. The global response rate consists of CR, PR, ORR, and DCR. The global CR was 1.26% (95% CI, 0.35–2.54; I^2^, 19.10), PR was 7.65% (95% CI, 3.32–13.37; I^2^, 76.40), ORR was 9.85% (95% CI, 4.40–16.95; I^2^, 81.60), and DCR was 18.33% (95% CI, 12.1827.59; I^2^, 79.20) **(A–D)**. 1-year overall survival rate and 6-months progression-free survival rate were 43.34% (95% CI, 35.70–51.15; I^2^, 68.70), and 17.24% (95% CI, 10.70–23.78; I^2^, 67.80) (EF).

The global response rate consists of CR, PR, ORR, and DCR. The global CR was 1.26% (95% CI, 0.35–2.54; I^2^, 19.10), PR was 7.65% (95% CI, 3.32–13.37; I^2^, 76.40), ORR was 9.85% (95% CI, 4.40–16.95; I^2^, 81.60), and DCR was 18.33% (95% CI, 12.18–27.59; I^2^, 79.20) (Figures 2A–D). 1-year overall survival rate and 6 months progression-free survival rate were 43.34% (95% CI, 35.70–51.15; I^2^, 68.70) and 17.24% (95% CI, 10.70–23.78; I^2^, 67.80) (Figures 2E,F).

#### Biomarker-Associated Analysis

**FIGURE 3:**
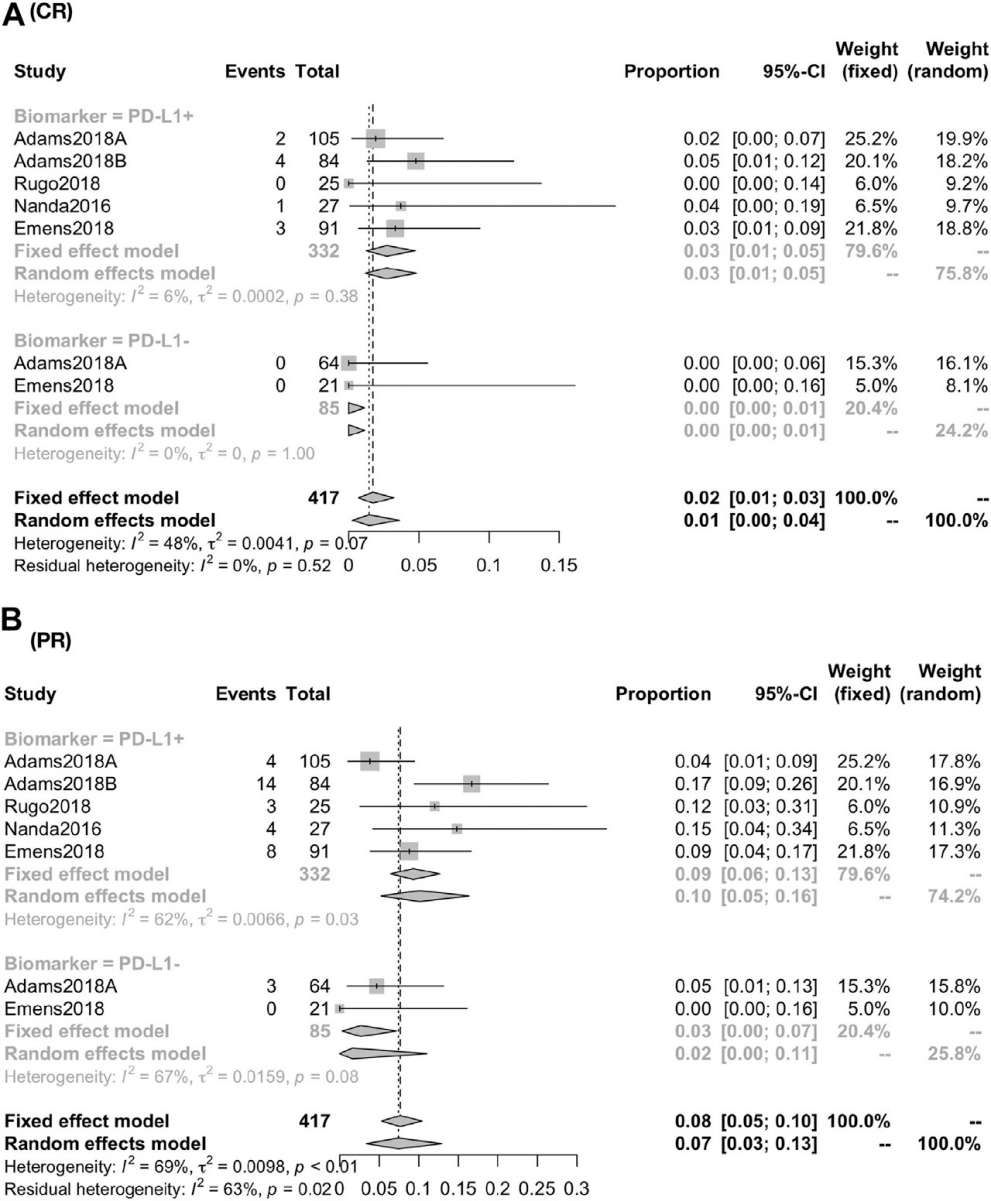
Global response rate between PD-1+ and PD-L1− for patients of CR **(A)** and PR **(B)**. The response rate was closely associated with the expression of PD-L1 biomarker (PD-L1+ vs. PD-L1−): the CR was 2.71% (95% CI, 1.24–4.72; I^2^, 05.00) vs. 0.00% (95% CI, 0.00–1.13; I^2^, 00.00); the PR was 9.93% (95% CI, 4.85–16.37; I^2^, 61.60) vs. 2.69% (95% CI, 0.01–8.03; I^2^, 66.70).

**FIGURE 4:**
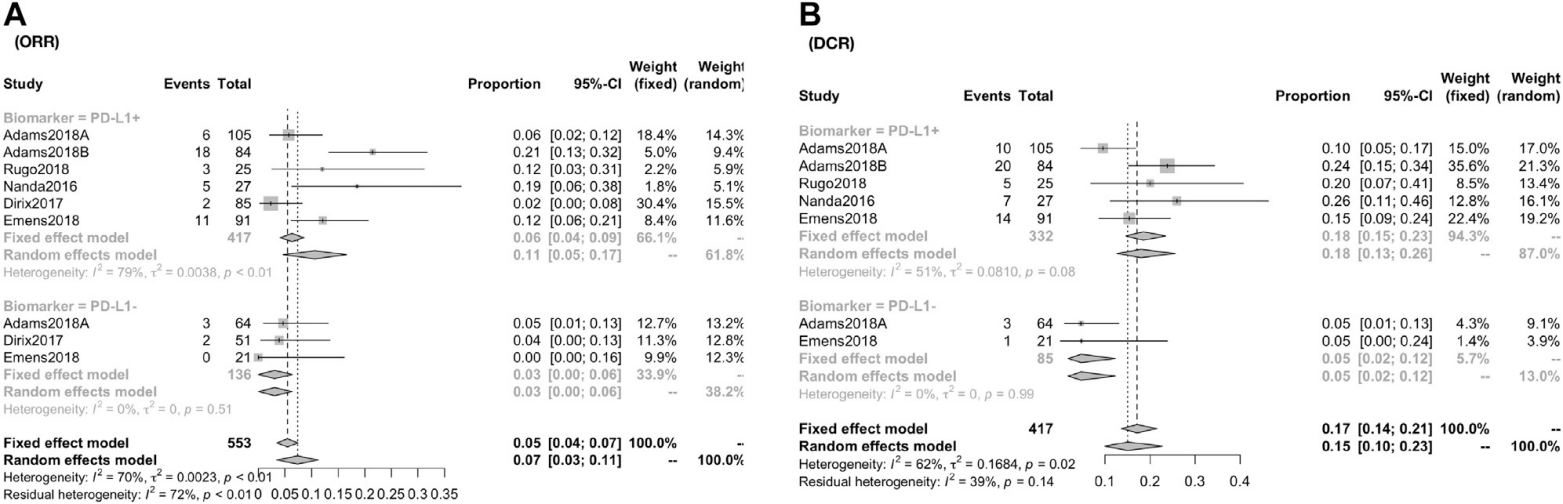
Global response rate and risk estimate between PD-1+ and PD-L1− for patients of ORR **(A)** and DCR **(B)**. The ORR was 10.62% (95% CI, 4.67–16.56; I^2^, 78.80) vs. 3.07% (95% CI, 0.00–6.43; I^2^, 0.00) [RR, 2.935 (1.189, 7.244)]; the DCR was 17.95% (95% CI, 12.61–25.55; I^2^, 51.30) vs. 4.71% (95% CI, 1.81–12.25; I^2^, 00.00) [RR, 3.584 (1.337, 9.608)].

The response rate was closely associated with the expression of PD-L1 biomarker (PD-L1+ vs PDL1−): the CR was 2.71% (95% CI, 1.24–4.72; I^2^, 05.00) vs. 0.00% (95% CI, 0.00–1.13; I^2^, 00.00); the PR was 9.93% (95% CI, 4.85–16.37; I^2^, 61.60) vs. 2.69% (95% CI, 0.01–8.03; I^2^, 66.70) (Figure 3); the ORR was 10.62% (95% CI, 4.67–16.56; I^2^, 78.80) vs. 3.07% (95% CI, 0.00–6.43; I^2^, 0.00) [RR, 2.935 (1.189, 7.244)]; the DCR was 17.95% (95% CI, 12.61–25.55; I^2^, 51.30) vs. 4.71% (95% CI, 1.81–12.25; I^2^, 00.00) [RR, 3.584 (1.337,9.608)] (Figure 4).

### Adverse Events

#### Global Incidence of AEs

**FIGURE 5:**
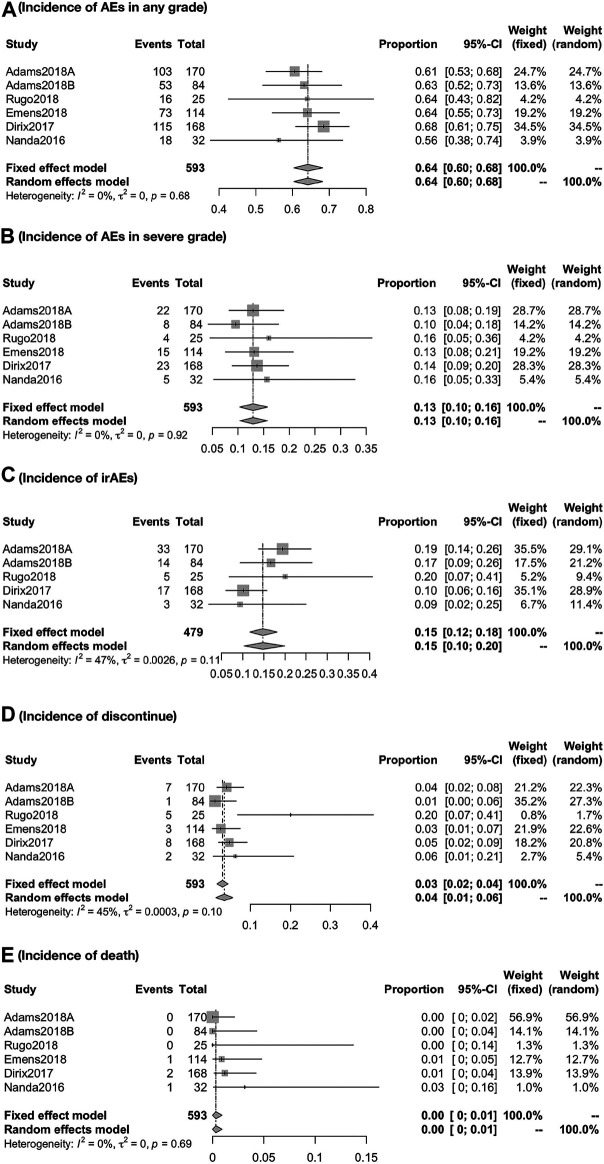
Global incidence of AEs in any grade **(A)**, in severe grade **(B)**, irAEs **(C)**, discontinue **(D)**, and death **(E)**. The overall incidence of AEs was 64.18% (95% CI, 60.43–68.17; I^2^, 0.00) in any grade and 12.94% (95% CI, 10.36–15.76; I^2^, 00.00) in severe grade. The incidence of irAEs was 14.75% (95% CI, 11.72–18.06; I^2^, 47.0). Besides, the incidence of discontinue and death due to treatment-related AEs was about 3.06% (95% CI, 1.68–4.44; I^2^, 45.50) and 0.31% (95% CI, 0.000.92; I^2^, 0.00), respectively.

Respectively, the overall incidence of AEs was 64.18% (95% CI, 60.43–68.17; I^2^, 0.00) in any grade and 12.94% (95% CI, 10.36–15.76; I^2^, 00.00) in severe grade. The incidence of irAEs was 14.75% (95% CI, 11.72–18.06; I^2^, 47.0). Besides, the incidence of discontinue and death due to treatment-related AEs was about 3.06% (95% CI, 1.68–4.44; I^2^, 45.50) and 0.31% (95% CI, 0.00–0.92; I^2^, 0.00), respectively (Figure 5).

#### Detail Incidence of AEs

**FIGURE 6:**
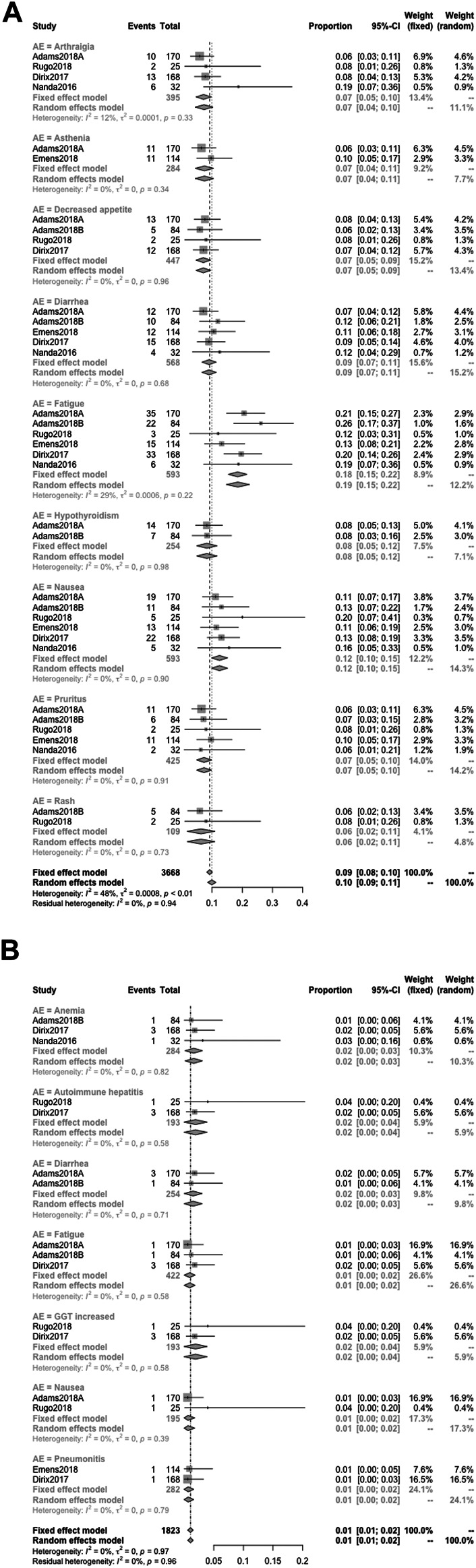
Detailed incidence of treatment-related AEs in any grade **(A)** and in severe grade **(B)**. When the specific AEs reported in at least two studies were analyzed, treatment-related AEs of any grade that occurred in at least 5% of patients were fatigue (18%), nausea (12%), diarrhea (9%), hypothyroidism (8%), arthralgia (7%), asthenia (7%), decreased appetite (7%), pruritus (7%), and rash (6%) **(A)**; treatment-related AEs of severe grade that occurred in at least 1% of patients were anemia (2%), autoimmune hepatitis (2%), diarrhea (2%), fatigue (1%), GGT increased (2%), nausea (1%), and pneumonitis (1%) **(B)**.

**FIGURE 7:**
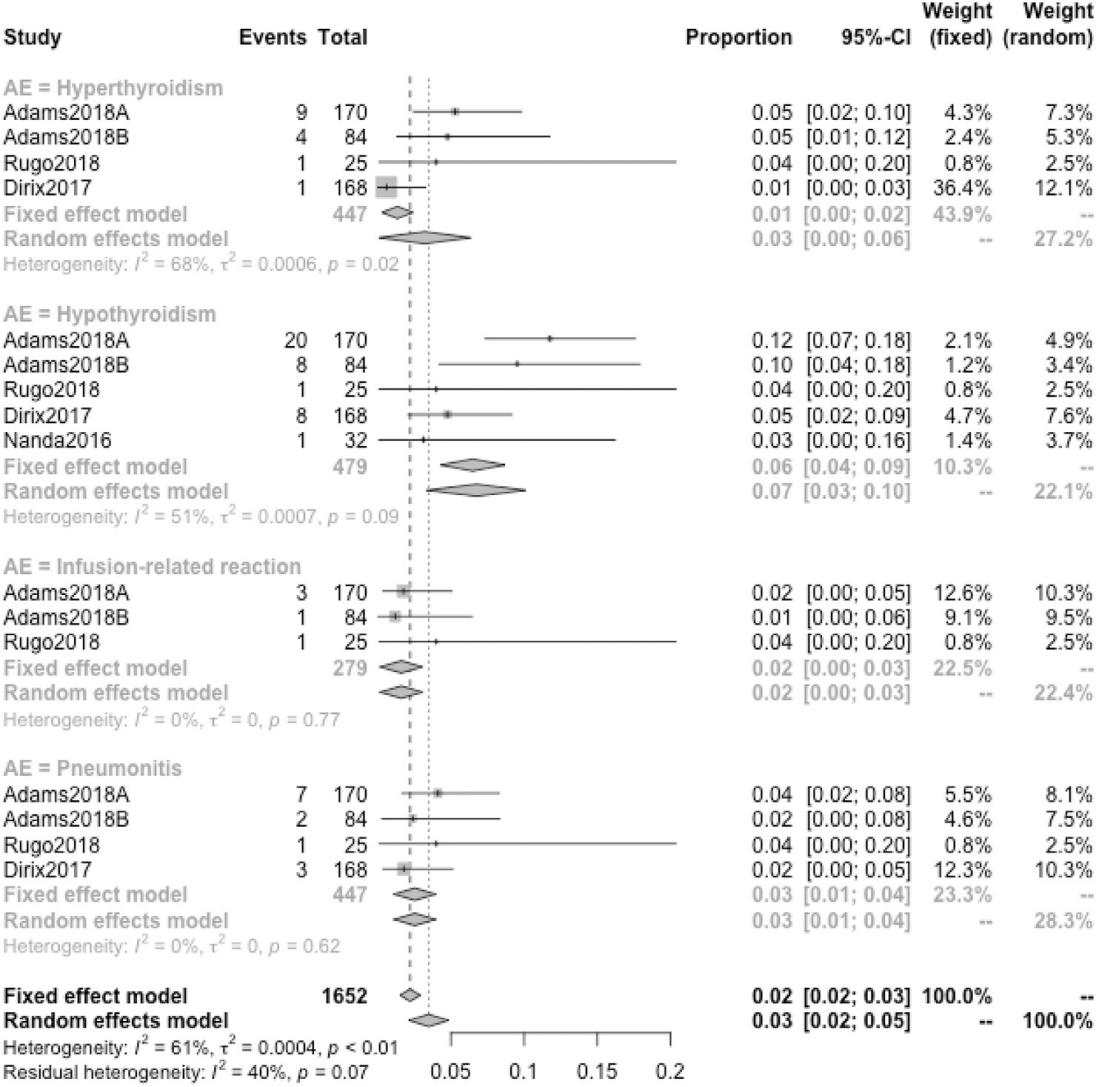
Detailed incidence of immune-related AEs in any grade. The primary irAEs were hypothyroidism (7%), hyperthyroidism (3%), and pneumonitis (3%) followed by infusion-related reactions (2%).

When the specific AEs reported in at least two studies were analyzed, treatment-related AEs of any grade that occurred in at least 5% of patients were fatigue (18%), nausea (12%), diarrhea (9%), hypothyroidism (8%), arthralgia (7%), asthenia (7%), decreased appetite (7%), pruritus (7%), and rash (6%) (Figure 6A); treatment-related AEs of severe grade that occurred in at least 1% of patients were anemia (2%), autoimmune hepatitis (2%), diarrhea (2%), fatigue (1%), GGT increased (2%), nausea (1%), and pneumonitis (1%) (Figure 6B); the primary irAEs were hypothyroidism (7%), hyperthyroidism (3%), and pneumonitis (3%) followed by infusion-related reaction (2%) (Figure 7).Quality Assessment and Publication Bias

Risk of bias graphs according to Cochrane Risk of Bias Tool was used to evaluate the methodological qualities of the studies. Blinding of participants and personnel was evaluated as a low-risk item because many studies were dose-escalation and single-arm trials. The overall risk of bias was evaluated as low risk. Therefore, the quality of the studies was satisfactory (Table 2). Finally, the Egger’s regression test showed no significant publication bias existed in ORR (Egger’s test *p* = 0.1551), DCR (Egger’s test *p* = 0.2853), and AEs in any grades (Egger’s test *p* = 0.2863).

**TABLE 2:** Risk of bias graph according to Cochrane risk of bias tool.

Study	Year	Randomization	Allocation concealment	Blinding of participants and staff^a^	Blinding of outcome assessors	Incomplete outcome data	Selective outcome reporting	Other sources of bias
AdamsA	2018	Low	Low	Low	Low	Low	Low	Low
AdamsB	2018	Low	Low	Low	Low	Low	Low	Low
Emens	2018	Low	Low	Low	Low	Low	Low	Low
Rugo	2018	Low	Low	Low	Low	Low	Low	Low
Dirix	2017	Low	Low	Low	Low	Low	Low	Low
Nanda	2016	Low	Low	Low	Low	Low	Low	Low
Kappa	NA	1.00	1.00	1.00	0.629	1.00	1.00	1.00

NA = not applicable.

a Blinding of participants and personnel was evaluated as low risk item because some studies were dose-escalation and single-arm trials. The overall risk of bias was evaluated as low risk.

## Discussion

In recent years, a myriad of innovations such as targeted drugs, chemotherapy, surgical techniques, and radiation have dramatically improved the treatment strategies as well as prognosis of malignancies. For early-stage breast cancer, survivorship is above 93% at 5 years (American Cancer Society, 2017). However, for lack of effective and tolerable treatment toward advanced breast cancer, survivorship is overwhelmingly disappointing and negative. The median overall survival time of MBC patients is up to 24 months (vary from months to years), while the median overall survival of mTNBC patients is just 8–13 months. Under this unfavorable scenario, success in finding anti-PD-1/PD-L1 agents is no doubt a good news for those patients suffering from advanced breast cancer.

**FIGURE 8:**
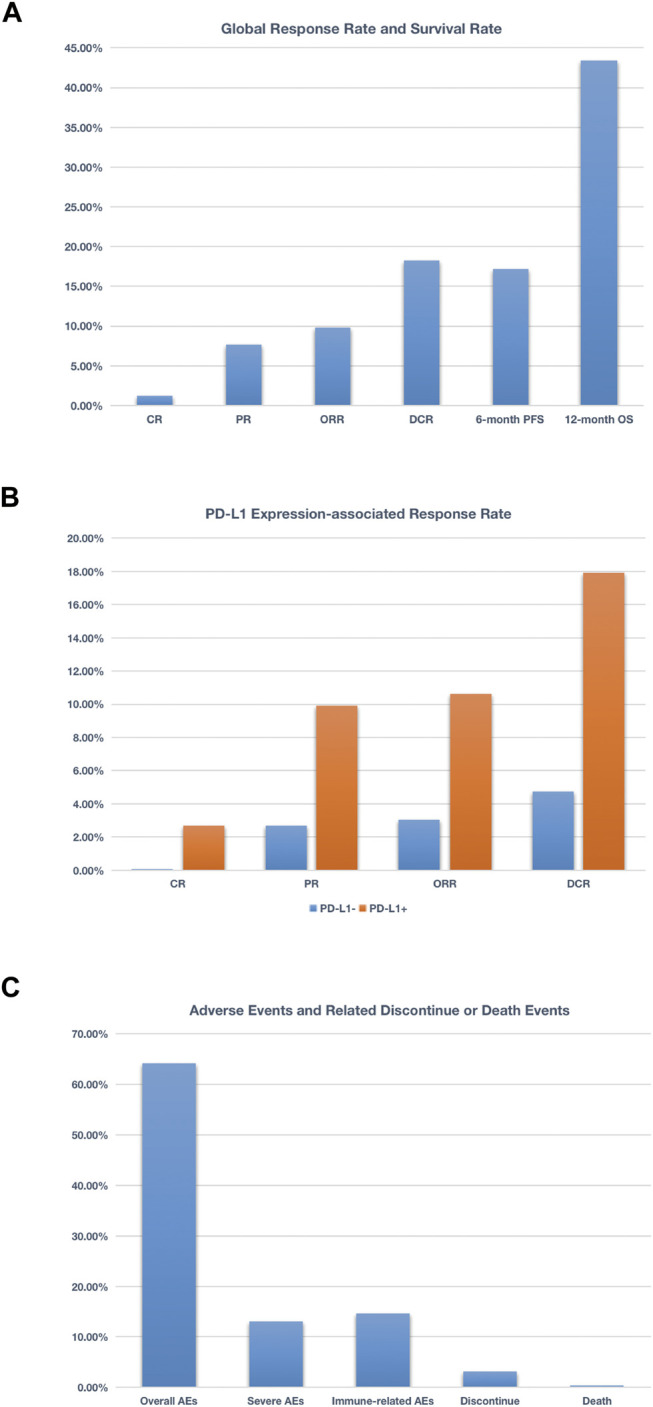
Schematic figure related to global response rate and survival rate **(A)**, schematic figure related to PD-L1 expression-associated response rate **(B)**, and schematic figure concerning adverse events and related discontinue or death events **(C)**. More than 17% patients could survive 6 months without disease progression, and over 43% patients could survive 1 year or more **(A)**; when compared PD-L1+ with PD-L1− patients, the CR was 2.52 vs 0.00%, the PR was 9.93 vs. 2.69%. Additionally, PD-L1+ patients even have approximately 3 times ORR and 3.6 times DCR of PDL1− patients **(B)**; nearly 65% patients treated with anti–PD-1/PD-L1 agents experienced at least one adverse event, and 13% patients suffered from at least one grade 3 or higher adverse event. As for irAEs, nearly 15% patients experienced one irAEs **(C)**.

This is the first meta-analysis report of the clinical efficacy and long-term safety associated with anti–PD-1/PD-L1 agents monotherapy in patients with metastatic breast cancer. Six relevant studies of high quality including 586 advanced breast cancer patients (454 TNBC patients and 132 patients with other types breast cancer) treated with anti–PD-1/PD-L1 agents were included in this meta-analysis. We observed that approximately one-tenth of patients received PD-1/PD-L1 inhibitors would achieve a partial or complete response, and more than one-sixth patients have achieved disease control. More than one-sixth patients could survive 6 months without disease progression, and over 43% patients could survive 1 year or more (Figure 8A). After analyzing the expression status of PD-L1, we found that when PD-L1–positive patients compared with global patients, similar ORR and DCR were observed (this may due to among 586 patients in this meta-analysis, 417 patients were at PD-L1–positive status), but when compared with PD-L1–negative patients, obvious differences do exist. The CR was 2.52 vs. 0.00% (PD-L1–positive vs. PD-L1–negative); the PR was 9.93 vs. 2.69%. Apart from that, we also observed that PD-L1–positive patients even have approximately three times ORR and 3.6 times DCR of PD-L1–negative patients (Figure 8B). Taken together, these data support the promising and durable effect of PD-1/PD-L1 inhibitors in patients who achieved a complete or partial response, and probably signal a more promising benefit in the group of patients with PD-L1–positive cancer types.

However, the selection of patients using PD-L1 assays is controversial mainly because of the absence of assay standardization: the PD-L1 staining patterns, cell patterns, scoring methods, and positivity thresholds are different in almost all of the cancer in which PD-L1 assay kits are approved by FDA, especially for triple-negative breast cancer. First, the cell patterns in which the assessment is evaluated differ from trials to trials. For example, KEYNOTE-119 is a phase III clinical trial, which used pembrolizumab as monotherapy vs. single-agent chemotherapy in advanced breast cancer setting (Winer et al., 2021). Researchers Cortes Javier et al. used CPS as primary end point and found no benefit in OS (Cortes J, Lipatov O, Im S-A, et al. ESMO, Barcelona, Spain; Sept 27– Oct 1, 2019), while TILs predict OS benefit to single agent pembrolizumab in KN119, contrary to PD-L1 (Sherene Loi et al., San Antonio Breast Cancer Conference 2019). Previous studies have confirmed that high tumor-infiltrating lymphocytes (TILs) were associated with response to PD-1/PD-L1 inhibitors in patients with breast cancer and a quantitative evaluation of TILs is important for any PD-L1 assay evaluation especially for breast cancer (Duchnowska et al., 2016; Hendry et al., 2017; Gonzalez-Ericsson et al., 2020). In this meta-analysis, we found that studies conducted by Rugo, 2018, and Nanda, 2016, assessed PD-L1 expression only in tumor cells, while the other four studies assessed that in both tumor cells and immune cells. The discrepancies of PD-L1 expression assessment can be found in Table 1, and all of these diversities may affect the results to some extent. Second, there are at least five non-equivalent assays for PD-L1, each with its own scoring system and tumor site indications. For breast cancers, FDA approved two assays: one was the Ventana PD-L1 (SP142) assay (Ventana Medical Systems, Tucson, AZ, United States) and cut-point (1% of TILs) from IMpassion130 trial in 2019, the other one was PD-L1 IHC 22C3 pharmDx assay (Agilent Technologies, Carpinteria, CA, United States) and its combined positivity score-scoring (CPS) system from the Keynote 355 breast cancer trial in 2020 (Salgado et al., 2020). However, the SP142 assay is poorly reproducible and is much less sensitive than other PD-L1 assays (Reisenbichler et al., 2020), which resulting different positive prevalence rates: in Impassion130, the prevalence of positivity using SP142 was 46%, while using 22C3 this was nearly 80% (Salgado et al., 2020), which might lead to incongruent clinical trial outcomes, lack of comparability and difficulty in seeking the best method to select patients for immunotherapy. Therefore, the current assay assessment should be updated. Previous studies have proven the prognostic and predictive importance of TILs assessment (Gonzalez-Ericsson et al., 2020; Brown et al., 2021). The systematic implementation of combined PD-L1 and TILs analyses as a comprehensive immuno-oncological biomarker to select patients for PD-1/PD-L1 inhibitor–based immune-therapy in breast cancer patients should be strongly considered by industry and academia (Gonzalez-Ericsson et al., 2020).

Recently, promising efficacy in TNBC has been reported for a combination therapy of atezolizumab administered in combination with nab-paclitaxel chemotherapy (NCT01633970) (Cyprian et al., 2019; Schmid et al., 2019). Earlier studies conducted by Adams S and Tolaney S in which the treatment regimen were atezolizumab combined with taxane chemotherapy (NCT01633970) and pembrolizumab combined with eribulin mesylate (NCT02513472), respectively, also showed a promising effect in TNBC patients (Tolaney et al., 2017). As for combined with targeted therapy, there were immunotherapy in combination with inhibitors of CDK4/6 (cyclin-dependent kinases 4 or 6), HER2-targeted therapy, poly (ADP-ribose) polymerase inhibitors, angiogenesis inhibitors, PARP inhibitors (niraparib or olaparib), etc. (Esteva et al., 2019). Apart from that, TAMs (tumor-associated macrophages) and Aurora-A inhibition also played the key role in regulating the activity of anti-PD-1/PD-L1 agents for breast cancer (Santoni et al., 2018; Yin et al., 2019). Therefore, based on PD-1/PD-L1 inhibitors, there are a variety of combination regimens to be discovered and applied to clinical practice.

As for adverse events, we found that nearly 2 in 3 patients treated with anti-PD-1/PD-L1 agents experienced at least one adverse event, and one-eighth patients suffered from at least one grade 3 or higher adverse event. As for irAEs, nearly one-sixth patients experienced one irAEs. Resulting from treatment-related AEs, about one in 30 patients discontinued the treatment, and the rate of death was 0.30% (3 cases reported died of liver failure, DIC, multiple organ metastasis, and respiratory distress) (Figure 8C). Based on these numbers, it is significant to provide relevant information to patients before they start treatment with an anti–PD-1 or PD-L1 agent.

**FIGURE 9:**
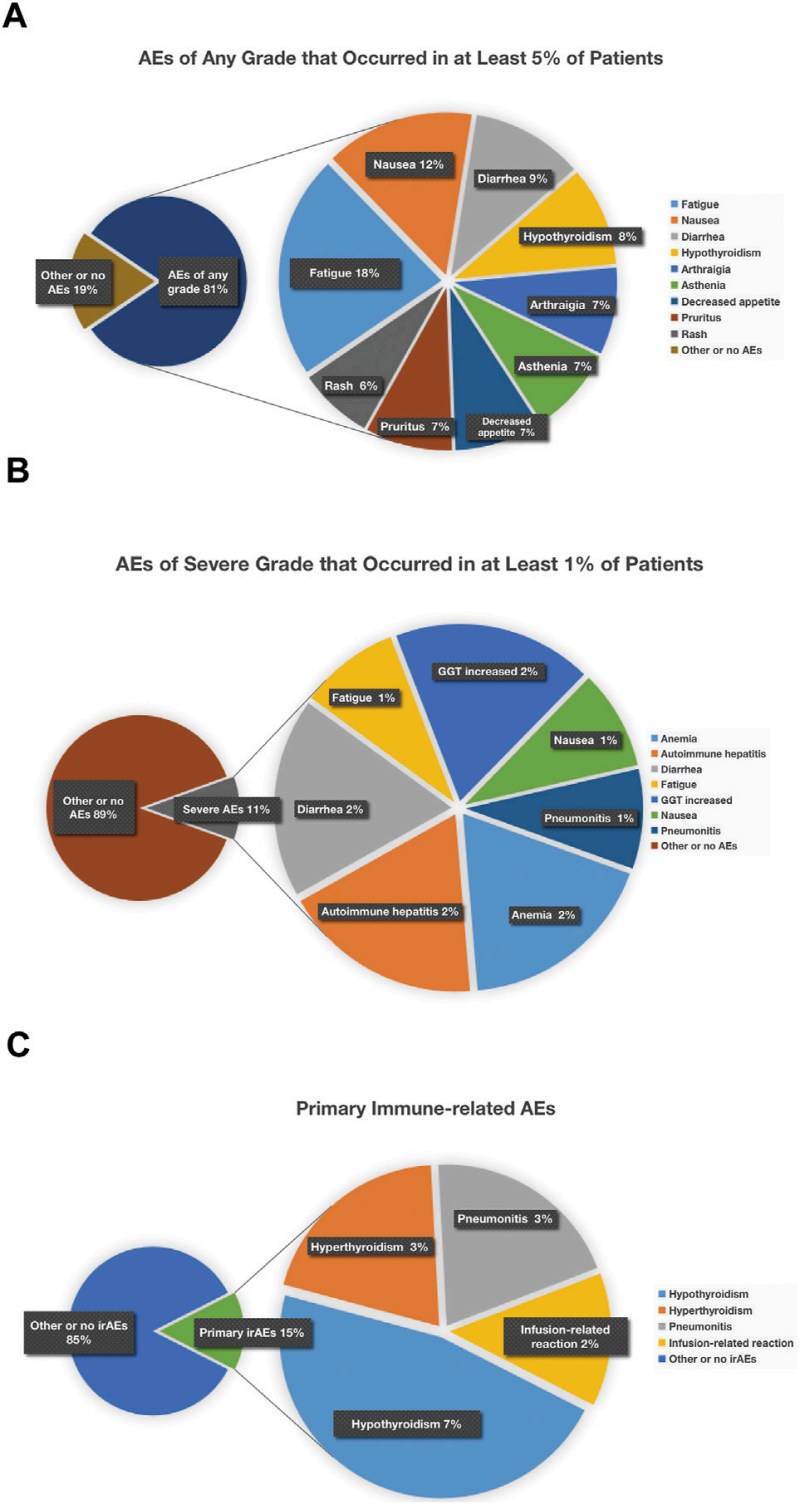
Treatment-related AEs of any grade that occurred in at least 5% of patients **(A)**, treatment-related AEs of severe grade that occurred in at least 1% of patients **(B)**, and chematic figure concerning primary immune-related AEs **(C)**. Treatment-related AEs of any grade that occurred in at least 5% of patients were fatigue (18%), nausea (12%), diarrhea (9%), hypothyroidism (8%), arthralgia (7%), asthenia (7%), decreased appetite (7%), pruritus (7%), and rash (6%) **(A)**; treatment-related AEs of severe grade that occurred in at least 1% of patients were anemia (2%), autoimmune hepatitis (2%), diarrhea (2%), fatigue (1%), GGT increased (2%), nausea (1%), and pneumonitis (1%) **(B)**; as for irAEs, manifesting as hypothyroidism (7%) was most common, and hyperthyroidism (3%), pneumonitis (3%), and infusion-related reaction (2%) were relatively less common **(C)**.

Fatigue was the most common all-grade adverse event, about one in five patients experienced it, but nearly one in 100 suffered a severe grade. Although it was less likely to be severe, given its relatively high incidence, it is worth disclosing to patients. Nausea, diarrhea, and hypothyroidism are the next most common all-grade adverse events (about one in 9–13), but the likelihood of patients experiencing severe manifestations of these adverse events is relatively low (≤1%) except diarrhea (approximately 2% patients in severe grade). Asthenia, decreased appetite, arthralgia, pruritus, and rash are the third-degree most common all-grade adverse events (about one in 14–16). For those adverse events, common but less likely to be severe, to inform patients is an essential part of dealing with these events (Figure 9A).

This meta-analysis also showed that the most common severe grade AEs (3 or higher adverse events) were immune-related, including anemia, autoimmune hepatitis, diarrhea, hepatic dysfunction, and pneumonitis (Figure 9B). According to several studies published, doctors found that pneumonitis was the most common cause of treatment-related death in patients treated with PD-1 and PD-L1 inhibitors. Apart from that, hepatitis was the most likely to be severe if it occurred, nearly half of hepatitis being grade 3 or higher. Diarrhea also occurred frequently and clinical vigilance is of vital importance for early recognition and intervention to prevent being serious colitis (Wang et al., 2017).

As for irAEs, manifesting as hypothyroidism (7%) was most common, while hyperthyroidism (3%), pneumonitis (3%), and infusion-related reaction (2%) were less frequent (Figure 9C). Usually these irAEs are temporary, but sometimes they can be severe or lethal. To reduce or alleviate these adverse reactions, it makes all the difference to monitor closely and recognize pertinent signs and symptoms early so as to provide proper management like prompt initiation of local or systemic immunologic suppression and so on, which is of huge benefit to improve outcomes for these patients. Generally speaking, to treat moderate or severe irAEs, it is necessary to discontinue current immunotherapy and start corticosteroids treatment in time.

Detailed therapeutic scheme is based on the severity of irAEs (Puzanov et al., 2017; Brahmer et al., 2018). For patients suffering moderate (grade 2) irAEs, doctors are suggested to suspend current immunotherapy, and are allowed to restart treatment when the symptoms or toxicity are alleviated (≤grade 1); and if the symptoms are not alleviated within 1 week, it is necessary to initiate glucocorticoid (prednisone 0.5 mg/(kgd) or other equivalent glucocorticoid). For patients suffering severe or life-threatening (grade 3 or 4) irAEs, doctors are suggested to suspend current immunotherapy permanently, and should start a high-dosage glucocorticoid (prednisone 1–2 mg/(kgd) or other equivalent glucocorticoid) treatment as soon as possible; when the symptoms are alleviated (≤grade 1), it is allowed to gradually decrease the dosage during at least 1 month. For those who showed the symptom of immune-related diarrhea, if it is not effective during the 3 days of glucocorticoid therapy, consider using infliximab (5 mg/kg), and for those patients who showed immune-related hepatitis, it is not allowed to use infliximab.

This meta-analysis has several limitations which should be addressed. First, the sample and data set in this meta-analysis was not adequate for statistical or visual examination of publication bias, furthermore the probable existence of such bias could not be well detected. We assume that this meta-analysis is subject to publication bias given that all of our analyses were based on publications. Second, the criteria for inclusion and exclusion in each study are different, which may affect the outcome and lead to heterogeneity among studies. In addition, this study is subject to any errors or biases of the original investigators; therefore, the results are generalizable only to patients eligible for these clinical trials. Third, there were discrepancies toward PD-L1 expression assessment and evaluation existing in different studies, which might be one of the sources of heterogeneity, and such diversities may also affect the results to some extent. Based on these limitations, we will continue to track this topic and update our further findings.

## Conclusion

In summary, this meta-analysis has revealed that durable antitumor clinical benefit can be achieved with anti-PD-1/PD-L1 monotherapy in a subset of patients with advanced breast cancer patients, and suggested that this treatment was generally well tolerated and had a manageable safety profile with most AEs at low grade. Survival was encouraging and promising, especially in patients with CR, PR, or SD status. Besides, we also found that the presence of PD-L1 can enrich for an advanced breast cancer population with higher response rate, thus further research and elucidation of the immunologic or molecular features of responders may identify a subset of patients who have excellent outcomes with anti–PD-1/PD-L1 monotherapy. On the basis of the results from these monotherapy clinical trials, scientists have conducted many studies of combination therapy, for example, immunotherapy combined with chemotherapy, which might produce higher value of therapeutic efficacy for metastasis breast cancer patients. We hope that this global overview of the adverse events of anti–PD-1/PD-L1 agents can serve as a reference by breast cancer clinicians and guide clinical practice.

## Data Availability

The original contributions presented in the study are included in the article/Supplementary Material, and further inquiries can be directed to the corresponding authors.
